# Acute and Prolonged Effects of Sweeteners and Sweetness Enhancers on Postprandial Appetite Sensations, Palatability, and Ad Libitum Energy Intake in Humans: A SWEET Sub-Study

**DOI:** 10.3390/nu18060948

**Published:** 2026-03-17

**Authors:** Sabina S. H. Andersen, Louise Kjølbæk, Jason C. G. Halford, Joanne A. Harrold, Anne Raben

**Affiliations:** 1 Department of Nutrition, Exercise and Sports, Faculty of Science, University of Copenhagen, 1958 Frederiksberg, Denmark; sabina@nexs.ku.dk (S.S.H.A.); louisekjoelbaek@nexs.ku.dk (L.K.); 2 Department of Psychology, University of Leeds, Leeds LS2 9JT, UK; j.halford@leeds.ac.uk; 3 Department of Psychology, University of Liverpool, Liverpool L69 7ZX, UK; harrold@liverpool.ac.uk

**Keywords:** non-caloric sweeteners, low-caloric sweeteners, hunger, satiety, sweet desire

## Abstract

**Background/Objectives:** Sweeteners and sweetness enhancers (S&SEs) have been proposed to potentially impair appetite regulation by stimulating sweet taste receptors beyond the perception of sweetness, similar to caloric sweeteners. The evidence is, however, not clear. **Methods**: This sub-study investigated the acute effects of a mixture of acesulfame potassium and cyclamate (Ace-K/Cyc) versus water on postprandial appetite sensations and energy intake at baseline, after a two-month weight loss period, and after a four-month weight loss maintenance period, including (S&SE group) or excluding S&SEs (Sugar group) in the diet. A total of 26 participants (18–65 years; BMI ≥ 25.0 kg/m^2^) were recruited from the one-year randomized controlled SWEET trial. Subjective appetite sensations were measured using visual analogue scales while fasting and nine times during a 250-min postprandial period. During this period, a standardized breakfast (0–10 min) was served and, 2 h later, a test drink containing either Ace-K/Cyc or water (120–130 min) was given. After 265 min, an ad libitum test meal was served. **Results:** Of 26 participants enrolled, 22 completed test day 2 and 16 completed test day 3. The S&SEs group rated lower prospective consumption and desire to eat something sweet after the test drink with Ace-K/Cyc compared to the sugar group consuming water (*p* < 0.05), with effects persisting after adjusting for taste. Initial differences in hunger were explained by taste palatability. This was true for all three test days. Ad libitum energy intake did not differ (*p* > 0.05). **Conclusions**: Ace-K/Cyc compared to water reduced feelings of prospective consumption and desire to eat something sweet acutely, after two months of weight loss, and after four months of weight loss maintenance. Due to the low sample size and power, larger studies are warranted to confirm these results.

## 1. Introduction

Non-caloric sweeteners (NCS) are food additives that possess the ability to sweeten food intensely without contributing significant energy [1,2]. Low-caloric sweeteners (LCS) are food additives that offer sweetness while containing a reduced amount of energy (on average 7.7 kJ/g) compared to e.g., sucrose (16.8 kJ/g) [1,3,4]. As a group, NCS and LCS can be defined as sweeteners and sweetness enhancers (S&SEs). These additives can enhance the palatability of food without the associated energy or glycemic impact [1,4,5,6,7]. Nevertheless, there is an ongoing debate regarding their potential effects on appetite regulation and energy metabolism [2,8,9,10,11,12,13,14].

The actuality of this debate is highlighted by the most recent recommendation by the World Health Organization (WHO) [15]. Based on a systematic review, WHO advises against using NCS for weight management, citing a lack of long-term benefits in relation to reducing body fat and suggesting potentially increased risks of non-communicable diseases associated with NCS [15,16]. In contrast, an expert panel’s consensus statements from 2020 concluded that NCS do not promote weight loss, do not negatively impact glucose regulation, and should be considered a strategy to reduce sugar intake [17]. The discrepancy between these recommendations likely stems from a difference in how the available evidence is weighted [18]. The WHO recommendation seems to give more weight to observational studies, while the expert panel’s consensus statements give higher weight to randomized controlled trials (RCTs) [15,17]. Since observational studies are prone to confounding and reverse causality, the WHO recommendation is conditional [15]. However, the contrast between these two recommendations clearly underlines the different results from RCTs and observational studies within this research field. This study contributes to this debate by examining both acute and prolonged effects of S&SEs on appetite regulation using a randomized controlled design, which provides higher-quality evidence than observational studies.

S&SEs have been proposed to potentially impair appetite regulation by stimulating sweet taste receptors beyond the perception of sweetness, similar to caloric sweeteners [8,9,11,12,14] and/or by disturbing the gut microbiome [10,13]. Furthermore, cephalic-phase response disruption [8,9,19] and neurological effects have also been suggested [8]. Mainly, NCS have been the focus of the sweet taste receptor theory [8,9,11,12,14]. If NCS can activate sweet taste receptors beyond the sensory taste of sweetness, they might temporarily stimulate carbohydrate oxidation, thus resembling the effect of carbohydrates [20]. However, as NCS normally cause a reduced amount of carbohydrates, especially when consumed as single drinks, the above may potentially create a situation with lower blood glucose levels. This could theoretically increase hunger [21,22,23]. Furthermore, NCS have been proposed to stimulate a preference for sweet foods and thereby sweet cravings, which both acutely and over time might change eating behaviors [8,9,19]. A perhaps unfavorable change in the gut microbiota over time due to the consumption of S&SEs might impair appetite by inducing, for example, insulin resistance [10,12,13]. Looking solely at long-term effects, repeated activation of cephalic-phase responses by NCS without the associated energy load has been proposed to impair appetite regulation by disrupting the hormonal and neurobehavioral pathways regulating appetite [8,9,19]. Lastly, for the NCS that are able to reach the blood brain barrier, it has been hypothesized that an accumulation of NCS could induce hippocampal damage, potentially promoting weight gain [8]. Evidence to support these hypotheses is mainly found in animal and in vitro studies, while data from human RCTs are either scarce or do not overall support these theories [8,9,10,12,13,24].

Looking at the evidence from human RCTs, some inconsistencies in ratings of appetite sensations (e.g., for hunger) after the consumption of NCS exist [11]. However, when evaluating energy intake, NCS seem to reduce energy intake both acutely and over time compared to sugar [25,26]. Furthermore, NCS seem to have similar effects to water on acute energy intake. However, results from longer-term studies are somewhat inconsistent [25,26]. Considering sweet preference, a systematic review from 2018 found that greater exposure to sweetened stimuli tended to lead to a lower preference for sweetness in short-term studies (<1 month); however, limited effects were found in longer-term studies (4–6 months) [27].

The aim of this sub-study was to investigate the effects of a mixture of acesulfame potassium and cyclamate (Ace-K/Cyc) on appetite sensations and energy intake compared to water. This was done before intervention (baseline), after two months of weight loss (WL), and after a four-month weight loss maintenance (WLM) period, with or without S&SEs in the diet. We also examined whether any observed effects could be attributed to differences in palatability between the test drinks.

## 2. Materials and Methods

### 2.1. Study Design and Participants

The sub-study was conducted from September 2020 to March 2022 (coinciding with the COVID-19 pandemic) at the Department of Nutrition, Exercise, and Sports, University of Copenhagen, Denmark. Participants were recruited from a 1-year RCT as part of the European Horizon 2020 project SWEET (Sweeteners and sweetness enhancers: Impact on health, obesity, safety and sustainability) (http://www.sweetproject.eu) [28]. The RCT aimed to determine if the prolonged consumption of S&SEs could lead to better 1-year WLM and improved obesity-related risk and safety markers compared to when S&SEs were excluded, both while consuming a healthy diet [28]. The main study, including the sub-study, was registered at www.clinicaltrials.gov (NCT04226911), approved by the National Research Ethics Committees (ref. H-19040679, 21 October 2019), 18 December 2019, and adhered to the Declaration of Helsinki. Informed consent forms were signed before any study-related activities.

**Figure 1:**
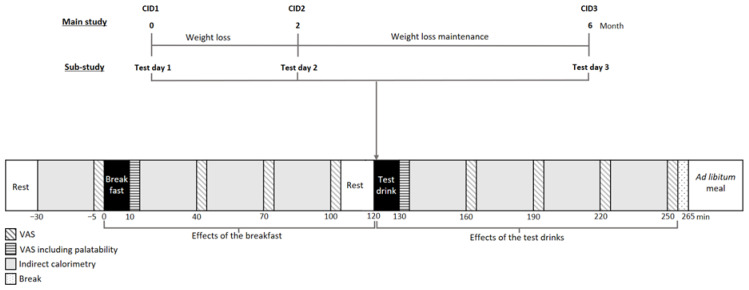
Study design of relevant parts of the main study and procedures in the sub-study. Abbreviations: CID, clinical investigation day; VAS, visual analogue scales.

The main study design and results have previously been published [28,29]. Both adults and families with overweight/obesity were recruited, but as only adults participated in this sub-study, the inclusion criteria for children are not presented here. The inclusion criteria for adults were an age of 18–60 years, body mass index (BMI) ≥ 25.0 kg/m^2^, and regular consumption of sugar-containing products. Exclusion criteria included chronic diseases or medications that could affect study outcomes (e.g., diabetes) [28]. The main study consisted of 4 clinical investigation days (CIDs) scheduled at baseline before WL (CID1, month 0), after WL (month 2), during WLM (month 6), and after WLM (month 12) (Figure 1). During the WL period, adult participants followed a low-energy diet (LED) aiming to reduce body weight by at least 5%. The LED included powdered shakes, soups, smoothies, and bars from the Cambridge Weight Plan (Northants, UK), allowing up to 4 products per day, providing 3347–4186 kJ/day [28]. After the WL period, participants followed a healthy diet, including the recommendation to reduce sugar to <10 E%. The strategy for the 2 intervention groups was either to include or exclude foods and drinks containing S&SEs, defined as the S&SEs or Sugar group, respectively [28]. Randomization was performed at a 1:1 ratio using blocks of 4 stratified by sex (female/male), age, and BMI, with a randomization list created by an independent person [28]. Participants were randomized after inclusion, but allocation was revealed to participants only after the WL period [28]. The participants did not receive reimbursement for taking part in this sub-study.

The sub-study aimed to include 48 Danish participants from the main study with an equal representation of sex and participants from each intervention group. It consisted of 3 additional test days: test day 1 (baseline): planned 7–10 days before month 0 (CID1); test day 2: planned 7–10 days after month 2 (CID2); and test day 3: planned 7 days before or after month 6 (CID3) (Figure 1). Test day 1 examined the acute effects of a standardized breakfast and a test drink with Ace-K/Cyc or water. Water was chosen to obtain a non-energy comparator differing only in sweetener content/sweet taste, without the added energy content from, for example, sucrose. On test day 2, the same investigation was conducted after participants had lost ≥5% of their body weight. Test day 3 explored the impact of a 4-month weight loss maintenance period of regular compared to no consumption of S&SEs as part of a healthy diet on the acute response to the breakfast and test drinks. Participants were blinded to the test drinks in the sub-study, while the investigators were not.

Prior to all test days, participants were told to fast ≥10 h and to avoid high-intensity physical activity, coffee, and smoking for 12 h. They were permitted to consume 0.5 L water during the fasting period. Each test day lasted approximately 6 h (8:00–14:00). Fasting body weight was measured, and for a subgroup (n = 15), a catheter was inserted into the elbow joint for blood sampling. After 15 min of bed rest, fasting substrate oxidation and energy expenditure were assessed by a ventilated hood system, appetite sensations and wellbeing recorded, and blood samples taken. A standardized breakfast was served at 0 min, and measurements repeated until 120 min. Subsequently, the test drink was served, and measurements continued until 250 min, followed by an ad libitum test meal at 265 min. A planned toilet break was allowed at 105 min (Figure 1). Data from the ventilated hood system and blood samples will be published separately.

Appetite sensations and wellbeing were assessed a total of 10 times per test day (Figure 1). Furthermore, the palatability of the breakfast and test drinks was assessed just after consumption of the meal/drinks (Figure 1). Appetite sensations, wellbeing, and palatability were measured using 100 mm electronic visual analogue scales (eVAS) [30], using a tablet and the program Evascale© version 1 (2017, Copenhagen, Denmark) [31,32]. The questions appeared in a random order to ensure participants’ attention to and consideration of each question. The questions for appetite sensations and wellbeing were: “How hungry do you feel?”; “How satisfied do you feel?”; “How full do you feel?”; “How much do you think you could eat?”; “Would you like to eat something sweet?”; and “Do you feel comfortable?”. The most negative and positive responses to the questions were anchored at each end of the 100 mm line, ranging from, for example, “I am not hungry at all” to “I have never been more hungry”, or from “I feel completely empty” to “I could not eat another bite” [30]. For palatability, participants were asked to assess the look, smell, taste, aftertaste, and general appearance of the breakfast/test drinks.

Total postprandial outcomes for appetite sensations and wellbeing were calculated via net incremental area over/under the curve (netAOC/AUC) using the trapezoidal rule. The netAOC/AUC was divided into 3 time periods, representing the effects of the breakfast, the test drinks, and the total time period (Figure 1). The time periods were: 0–100 min, 100–250 min, and 0–250 min. The 100-min time point was used as the fasting measurement when calculating the 100–250 min netAOC/AUC.

### 2.2. Test Meals

The standardized breakfast included Arla Cultura^®^ (sour yogurt) with oat and cranberries and 250 g water (Arla Foods, Viby, Denmark) (Table 1). Participants in the S&SEs group received a test drink with 5 g of Atwell^®^-0-calories^®^ (equal to a sweetness intensity of 50 g sugar) and 400 g water (Toft Care A/S, Roslev, Denmark). Atwell^®^ contains a mixture of Ace-K and Cyc. Participants in the sugar group received 405 g of pure tap water. Participants were instructed to consume the meal/drink over 10 min, meaning that the last bite/sip should be consumed in the 10th minute.

The ad libitum test meal consisted of gluten-free pizza (Ristorante Pizza Mozzarella) from Dr. Oetker© (Dr. Oetker© Danmark A/S, Glostrup, Denmark), chosen for its uniformity and general acceptance (Table 1). Male participants were served 3 pizzas and females were served 2 pizzas on a tray with a scissor and 250 g of water. The scissor was intended to encourage participants to actively choose and cut each new piece of pizza, rather than having pre-cut slices readily available. The participants were instructed to not leave behind the pizza crust, eat until pleasantly satiated, and drink all the water. Talking was not allowed while consuming any of the test meals/drinks.

### 2.3. Statistical Analysis

Sample size estimation was based on the primary outcome, which was fat oxidation. Statistical analyses were performed in R version 4.2.1 (R Core Team, 2022, Vienna, Austria). Initial data cleaning involved a visual inspection of individual curves, and no outliers (data outside ±3 SD). Appetite sensations and wellbeing were analyzed both as repeated measurements and netAOC/AUC via mixed linear ANCOVA models. Models for repeated measurements included interactions between meal, test day, and time, and were adjusted for participant number and test day as random effects, and age, sex, smoking status, BMI at baseline, weight change from baseline, and fasting value (0 min) of the given outcome as fixed covariates. In case of a time-meal-test day interaction, time-meal interaction, or meal effect, time points and estimated mean differences were compared using emmeans (estimated marginal means) in R. Models for netAOC/AUC included interactions between meal, test day, and period, and were adjusted for participant number and test day as random effects, and age, sex, smoking status, BMI at baseline, and weight change from baseline as fixed covariates.

Ad libitum energy intake and palatability were also analyzed using mixed linear ANCOVA models, including interactions between meal and test day, and adjusted for participant number as random effect, and age, sex, and smoking status as fixed covariates. Additionally, the model for ad libitum energy intake included BMI at baseline and weight change from baseline as fixed covariates. When differences were found, Holm’s method was used to adjust for multiple testing to counteract the problem of multiple comparisons. Models including weight change from baseline were run without this covariate to ensure that any effect caused by the weight loss combined with Ace-K/Cyc was not masked. This did not change the results; thus, only results including weight change from baseline as a covariate are shown. In addition to this, models for appetite sensations and wellbeing were rerun, including the palatability score taste as a fixed covariate, as the test drink with Ace-K/Cyc was found to be less tasty on all 3 test days compared to the test drink with water. Taste scores for the breakfast were included as a covariate for the 0–100 min netAOC/AUC and for the time points 10 to 100 min when analyzing repeated measurements. Taste scores for the test drinks were included as a covariate for the 100–250 min and 0–250 min netAOC/AUC, as well as for the time points 130 to 250 min when analyzing repeated measurements. Adjusting for taste in this way means that any differences in appetite sensations caused by differences in taste perception are removed. Model validation was checked by normal quantile, and residual plots, and all models were deemed valid. Available case analyses were chosen due to high dropout rates. Two participants were served the wrong test drink on test day 1, but received the correct test drink going forward. In the analyses, their data were evaluated based on the test drink they received on each specific test day. When calculating baseline characteristics (means ± SD), these 2 participants were included in their original group. Figures show unadjusted means ± standard error mean (SEM). The significance level was *p* < 0.05.

## 3. Results

### 3.1. Study Flow

**Figure 2:**
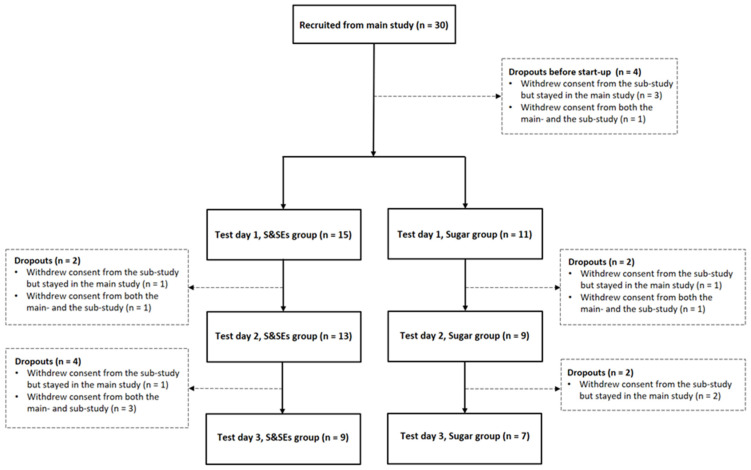
Flow chart of participants throughout the sub-study. Abbreviations: S&SEs, sweeteners and sweetness enhancers.

Thirty participants were recruited for the sub-study: 26 completed test day 1 (month 0); 22 completed test day 2 (month 2); and 16 completed test day 3 (month 6). Dropout occurred due to, for example, demanding study activities, discomfort in the ventilated hood, or personal reasons (Figure 2).

### 3.2. Baseline Characteristics

There were no differences in baseline characteristics between the S&SEs and sugar group (Table 2) or between completers and dropouts; all *p* > 0.05.

### 3.3. Fasting Measurements

**Figure 3:**
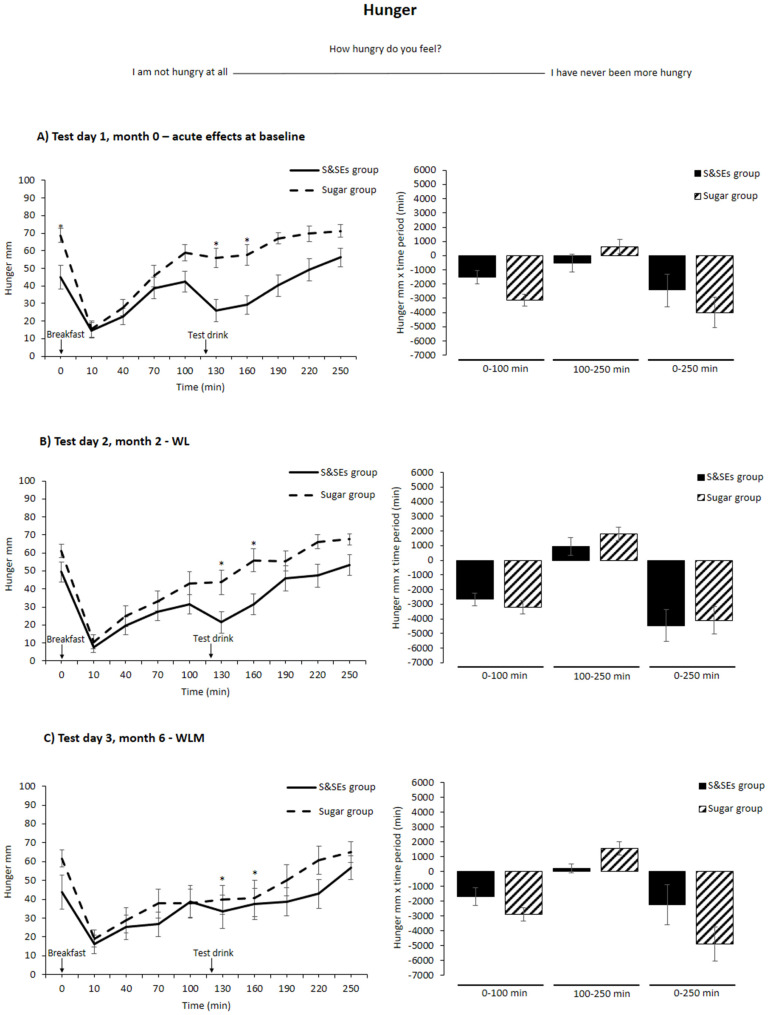
Hunger as unadjusted means ± SEM at different time points (**left**) and netAOC (**right**) for all 3 test days: (**A**) test day 1, month 0—acute effects at baseline; (**B**) test day 2, month 2—WL; and (**C**) test day 3, month 6—WLM. To illustrate that netAOC provides information about how much hunger is inhibited, the netAOC means have been reversed. ANCOVA models were used to test for differences between groups. Holm’s method was used for multiple testing adjustments, and differences after adjustment are indicated with an asterisk (*, *p* < 0.05). Abbreviations: SEM, standard error mean; S&SEs, sweeteners and sweetness enhancers; WL, weight loss; WLM, weight loss maintenance.

No differences were found between the groups in fasting ratings of fullness, satiety, desire to eat something sweet, or prospective consumption after adjusting for multiple testing (all *p* > 0.05). However, the S&SEs group exhibited lower fasting hunger on test day 1 compared to the sugar group (20.6 ± 7.6 mm, *p* = 0.03). No differences were observed on test days 2 and 3 (*p* > 0.05) (Figure 3). For all five appetite sensations, no differences were found between the groups when comparing changes in fasting measurements between the three test days (*p* > 0.05). No differences in fasting wellbeing were found on any test day or between test days (*p* > 0.05).

### 3.4. Subjective Appetite Sensations

No time-meal-test day interaction, time-meal interaction, or meal effect was found for fullness and satiety after adjusting for multiple testing (all *p* > 0.05). Furthermore, netAUC fullness and satiety did not differ between the two groups for any of the time periods (0–100 min, 100–250 min, 0–250 min) on any test day, or when comparing changes in netAUC between the three test days (all *p* > 0.05). Rerunning analyses with taste as a covariate did not change the results (all *p* > 0.05).

For hunger, no time-meal-test day interaction was found (*p* = 0.12); however, a time-meal interaction was found across the three test days (*p* < 0.001) (Figure 3). Post-hoc analyses showed that the S&SEs group felt less hungry compared to the sugar group after the test drink (time points: 130 min, 16.1 ± 5.2 mm, *p* = 0.03 and 160 min, 15.3 ± 5.2 mm, *p* = 0.03) after adjusting for multiple testing. For netAOC hunger, no difference between groups was found for any of the time periods on any test day or between the three test days (*p* > 0.05) (Figure 3). When rerunning analyses with taste as a covariate, no difference between the groups was found at time points 130 min (*p* = 0.06) and 160 min (*p* = 0.08), after adjusting for multiple testing.

**Figure 4:**
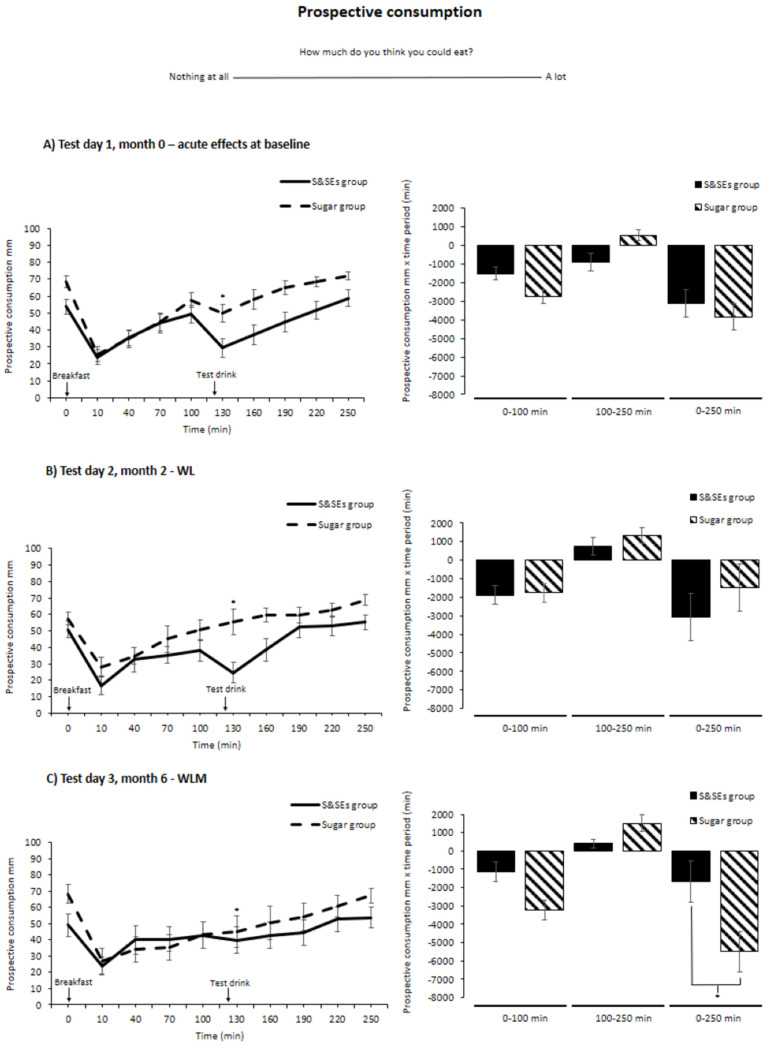
Prospective consumption shown as unadjusted means ± SEM at different time points (**left**) and netAOC (**right**) for all three test days: (**A**) test day 1, month 0—acute effects at baseline; (**B**) test day 2, month 2—WL; and (**C**) test day 3, month 6—WLM. To illustrate that netAOC provides information about how much prospective consumption is inhibited, the netAOC means have been reversed. ANCOVA models were used to test for group differences. Holm’s method was used for multiple testing adjustments, and differences after adjustment are indicated with an asterisk (*, *p* < 0.05). Abbreviations: SEM, standard error mean; S&SEs, sweeteners and sweetness enhancers; netAOC, net incremental area over the curve; WL, weight loss; WLM, weight loss maintenance.

For prospective consumption, no time-meal-test day interaction was found (*p* = 0.33); however, a time-meal interaction was found across the three test days (*p* < 0.001) (Figure 4). Post=hoc analyses showed that the S&SEs group rated lower prospective consumption compared to the sugar group right after the test drink (time point: 130 min, 16.4 ± 5.5 mm, *p* = 0.03) after adjusting for multiple testing. Contrary to this, the S&SEs group showed a lower inhibition of prospective consumption for the 0–250 min netAOC compared to the sugar group on test day 3 (3505 ± 1210 mm × 250 min, *p* = 0.04) after adjusting for multiple testing. No other differences were found on any test day or when comparing changes between test days for netAOC (all *p* > 0.05) (Figure 4). However, when rerunning the analyses with taste as a covariate, there was no difference between the groups for the 0–250-min netAOC (*p* = 0.055), after adjusting for multiple testing.

**Figure 5:**
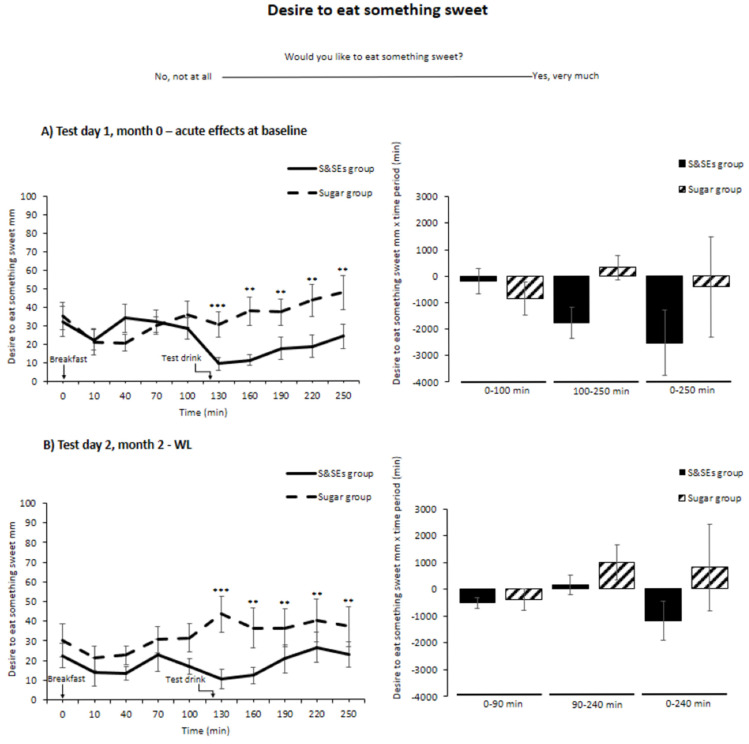

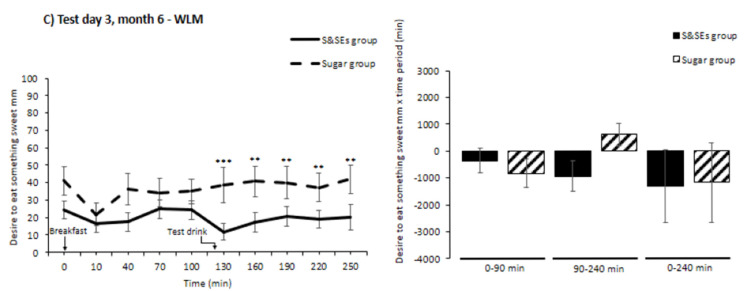
Desire to eat something sweet shown as unadjusted means ± SEM at different time points (**left**) and netAOC (**right**) for all 3 test days: (**A**) test day 1, month 0—acute effects at baseline; (**B**) test day 2, month 2—WL; and (**C**) test day 3, month 6—WLM. To illustrate that netAOC provides information about how much desire to eat something sweet is inhibited, the netAOC means have been reversed. ANCOVA models were used to test for differences between groups. Holm’s method was used for multiple testing adjustments, and differences after adjustment are indicated with an asterisk (**, *p* < 0.01; ***, *p* < 0.001). Abbreviations: SEM, standard error mean; S&SEs, sweeteners and sweetness enhancers; WL, weight loss; WLM, weight loss maintenance.

Regarding desire to eat something sweet, no time-meal-test day interaction was found (*p* = 0.19), but a time-meal interaction was found across the three test days (*p* < 0.001) (Figure 5). Post-hoc analyses showed that the S&SEs group rated a lower desire to eat something sweet compared to the sugar group at all five time points after the test drink (time points: 130 min, 27.5 ± 5.6 mm, *p* < 0.001; 160 min, 25.7 ± 5.6 mm, *p* = 0.001; 190 min, 19.0 ± 5.6 mm, *p* = 0.005; 220 min, 20.0 ± 5.6 mm, *p* = 0.003; and 250 min, 20.8 ± 5.6 mm, *p* = 0.002), after adjusting for multiple testing (Figure 5). No differences were seen for netAOC (all *p* > 0.05) after adjusting for multiple testing. Rerunning analyses with taste as a covariate found, in addition to the time points above, a reduced desire to eat something sweet across the three test days for the S&SEs group compared to the sugar group just before serving the test drink (time point: 100 min, 14.8 ± 5.5 mm, *p* = 0.03).

### 3.5. Palatability Scores, Wellbeing, and Ad Libitum Energy Intake

For the standardized breakfast, none of the five palatability measures differed between groups on any test day or when comparing changes between test days (all *p* > 0.05).

**Figure 6:**
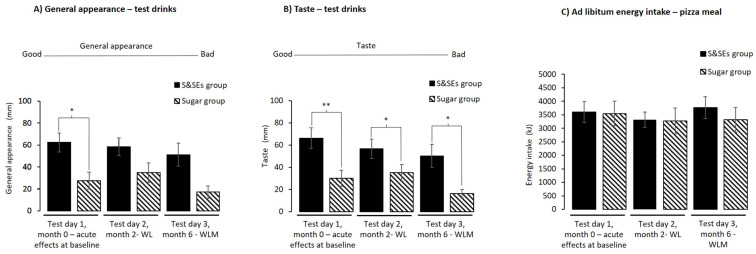
Palatability measures and ad libitum energy intake shown as unadjusted means ± SEM for all 3 test days: (**A**) general appearance—test drinks; (**B**) taste—test drinks; (**C**) ad libitum energy intake—pizza meal. For the ad libitum meal, data from one participant were excluded on test day 2 due to an error in the kitchen laboratory. ANCOVA models were used to test for differences between groups. Holm’s method was used for multiple testing adjustments and differences after adjustment are indicated with an asterisk (*, *p* < 0.05, **, *p* < 0.01). Abbreviations: SEM, standard error mean; S&SEs, sweeteners and sweetness enhancers; WL, weight loss; WLM, weight loss maintenance.

For the two test drinks, look, smell, and aftertaste did not differ between groups on any test day or when comparing changes between test days (all *p* > 0.05). For general appearance, the test drink with Ace-K/Cyc was found to be less appealing than water on test day 1 (29.1 ± 11.6 mm, *p* = 0.045) after adjusting for multiple testing (Figure 6A). No differences were found when comparing changes between test days (all *p* > 0.05). For taste, the Ace-K/Cyc drink was found to be less tasty on all three test days (test day 1: 37.5 ± 11.8 mm, *p* = 0.008; test day 2: 28.3 ± 12.8 mm, *p* = 0.03; test day 3: 39.1 ± 14.5 mm, *p* = 0.02) (Figure 6B) after adjusting for multiple testing. No differences were found when comparing changes between test days (all *p* > 0.05). Furthermore, there were no differences in wellbeing for any of the performed analyses (all *p* > 0.05) after adjusting for multiple testing.

Ad libitum energy intake did not differ between groups on any test day (Figure 6C) or when comparing changes between test days (all *p* > 0.05).

## 4. Discussion

### 4.1. Summary of Findings

The S&SEs group rated lower hunger, prospective consumption, and desire to eat something sweet at different time points across the three test days after the test drink with Ace-K/Cyc compared to the sugar group consuming water. The S&SEs group rated a lower inhibition of prospective consumption for the 0–250 min netAOC on test day 3 compared to the sugar group. However, when including taste as a covariate, the group differences for hunger and 0–250 min netAOC prospective consumption disappeared. Ratings of fullness, satiety, and ad libitum energy intake did not differ between groups or test days. Four months on, the intervention diets did not change the response to a standardized breakfast, to a test drink, or the ad libitum energy intake, thus indicating lasting effects without adaptations over time. Still, the low power and simple size warrant caution when interpreting these data.

### 4.2. Appetite Sensations and Energy Intake

The findings of no effects of Ace-K/Cyc on fullness, satiety, and hunger (after adjusting for taste) and a decreased effect on prospective consumption compared to water contradict older research and prevailing myths, which demonstrated a phenomenon defined as rebound hunger [11,33]. Rebound hunger was demonstrated in an acute study by Rogers et al. [33]. Here, the consumption of drinks containing aspartame or saccharine initially led to suppressed appetite ratings for hunger, prospective consumption, desire to eat, and increased feelings of fullness. However, within an hour, these ratings increased above the baseline level compared to water (rebound hunger). Notably, only the aspartame drink showed significant effects compared to water and only for hunger and desire to eat. The study also included a test drink with Ace-K. Here, Ace-K did not exhibit initial appetite suppression but followed the pattern of increases above baseline after consumption compared to water; however, these changes were not statistically significant. No differences in energy intake were observed 1 h after consuming the NCS drinks compared to water [33]. Methodological differences could potentially explain the discrepancy: (1) Rogers et al. [33] used pure Ace-K, aspartame, or saccharine, while we used an Ace-K/Cyc blend; (2) their study had a shorter follow-up (1 h vs. 4+ h); (3) our participants had overweight/obesity vs. normal weight in Rogers et al. [33]; and (4) the statistical power, sample size, and analysis methods differed. The appetite-increasing effects of aspartame on hunger compared to water have, however, not been demonstrated in many subsequent acute pre-load studies [34]. Most of these pre-load studies simply show effects on hunger similar to water [34], a finding also supported by more recent acute studies, including isoenergetic conditions evaluating a range of NCS on appetite ratings [35,36,37,38]. Thus, these results align with the findings of this study.

Longer-term studies evaluating these subjective appetite sensations, comparing NCS to either an isoenergetic or a non-isoenergetic comparator, show generally similar ratings of subjective appetite sensations between the NCS and the comparator [36,39,40,41,42]. This supports the finding of this study, namely, that four months of regular consumption of S&SEs in the diet did not affect the response to a standardized breakfast compared to a diet with no consumption of S&SEs.

When analyzing the data with the originally planned model, this study showed a reduced hunger up to 40 min after the Ace-K/Cyc test drink compared to water across the three test days. Reanalyzing the results with taste as a covariate suggested that palatability differences between the test drinks account for these findings. However, since the taste disparity is mainly due to sweetness, including taste as a covariate may obscure any potential sensory impact of sweetness on hunger. The same argument can also be used for prospective consumption, where the difference between groups on test day 3 for the 0–250 min netAOC prospective consumption disappeared after including taste as a covariate. Thus, it is difficult to determine whether different palatability of the test drinks or the sweet taste itself caused the originally seen differences between the groups. This clearly demonstrates challenges that are hard to overcome when testing NCS versus water on appetite.

Looking specifically at the repeated measurements analyses for prospective consumption, this study’s results directly contrast with those of the aforementioned study by Rogers et al. [33]. Thus, this study found a significant reduction in prospective consumption 10 min after the Ace-K/Cyc drink, whereas Rogers et al. [33] found no significant reduction for any of the test drinks. An explanation for this might be differences in dose of NCS and the fact that this study included a mixture of Ace-K/Cyc and not pure Ace-K, aspartame, or saccharine.

Comparisons between the results of this study and non-isoenergetic studies on NCS drinks versus sugar-rich drinks should be made with caution, as the energy disparity between interventions can be a confounding factor. Thus, a recently published acute study by Almiron-Roig et al. [43] showed that a test drink containing sucralose/Ace-K resulted in a temporal profile with higher ratings of prospective consumption compared to sucrose when consumed 7 min prior to a standardized breakfast. However, this did not translate into a variation in 24-h energy intake. While the contrast between these two groups could be explained by the energy disparity between the test drinks, other blends of NCS did not exhibit any variation in prospective consumption compared to sucrose. The results of the study by Almiron-Roig et al. [43] therefore indirectly contrast the results of this study. However, NCS blend and design differences might explain these contrasting results.

Aligning with the results of our study is a ten-week ad libitum study by Sørensen et al. [41]. After ten weeks, a diet containing foods and drinks with a mix of NCS (54% aspartame, 23% Cyc, 22% Ace-K, and 1% saccharin) resulted in higher levels of fullness and lower prospective consumption at different time points after lunch and dinner compared to a diet with sugar. This was despite a lower ad libitum energy intake at lunch and dinner in the NCS group.

This study’s findings of reduced desire to eat something sweet could be attributed to the term sensory-specific satiety, where exposure to sweetness, for example, leads to a decrease in the perceived pleasantness and preference for foods/drinks sharing the same attribute [27]. That NCS can increase sensory-specific satiety is in agreement with previous acute studies comparing NCS to water [36,44].

In a paper by Rogers et al. [44], three acute studies were conducted. In the first study, the effects of a drink containing sucralose versus water were evaluated on parameters such as the desire to consume and the pleasantness of taste. These parameters were assessed shortly (within 1 min) before and after consuming the two test drinks. The second study had a similar design; however, it expanded the evaluation to include another round of assessments 2 h after consuming the test drinks. In the third study, participants consumed sugar- or NCS-sweetened (aspartame) coca cola, or still or carbonated water shortly before and during ad libitum consumption of sweet and non-sweet foods. Together, the results of these three acute studies showed that drinks with NCS acutely decreased desire for and intake of sweet foods, but not total ad libitum energy intake [44]. In addition, an acute and longer-term study by Fantino et al. [36] partly supports these results. In the acute part of the study, intake of sweet foods in a laboratory setting was lowered when consuming an NCS lemonade (a mix of Ace-K, aspartame, and sucralose) compared to water for female participants, while no difference was found for male participants. In the longer-term part of the study, consuming NCS lemonade for five weeks did not affect energy intake, micronutrient intake, or the selection of sweet foods compared to water [36]. In line with this, a study by Appleton et al. [45] found no difference between groups on ratings of pleasantness of sweet foods and intake of sweet foods when being exposed to a breakfast sweetened with sucralose compared to a non-sweet, isoenergetic breakfast for three weeks. These findings were recently supported by the large RCT “Sweet Tooth” including 180 healthy volunteers [46]. Here, sweet taste preference was not affected by high, medium, or low exposure to sweet-tasting foods for six months. That NCS compared to water do not increase preference for sweet foods is further supported by a longer-term study by Popkin et al. [47]. They found that consumption of NCS in the diet for six months compared to water led to a reduction in energy intake from sugars and desserts [47]. From a practical standpoint, the reduced desire for sweet foods following sweetener consumption could support weight management efforts by decreasing sweet food intake, though this was not directly measured in our ad libitum test meal, which consisted of savory pizza.

In total, despite the different study designs, all the above studies and our own findings do not support the notion that NCS will increase preference for sweet foods acutely or in the longer term.

This study found that the acute effects of Ace-K/Cyc on ad libitum energy intake were comparable with water both at baseline and after a period with regular consumption of S&SEs. This is in line with the acute findings in the aforementioned systematic review by Rogers et al. [25] and a more recent study comparing different sweeteners and water on ad libitum energy intake [48]. Due to the design of this study, it is difficult to compare it to longer-term studies assessing energy intake in relation to NCS and/or LCS. However, the results do not indicate an effect on the ad libitum test meal after exposure to S&SEs for four months compared to no consumption of S&SEs in the diet.

### 4.3. Strengths and Limitations

This study excels in its distinctive capacity to evaluate Ace-K/Cyc’s acute effects on appetite both before and after regular consumption of S&SEs in the diet, as well as its ability to investigate the impact of regular consumption of S&SEs on responses to a standardized breakfast and an ad libitum test meal. This was achieved by using water as the control, incorporating a standardized breakfast and an ad libitum test meal, conducting tests before and after four months on a diet with or without S&SEs, and dividing the 4-h netAUC/AOC into three distinct time periods to differentiate between the effects of the standardized breakfast and the test drinks.

However, a limitation of the study is the low sample size and low statistical power. Power was calculated based on the primary outcome of the sub-study, which was fat oxidation.To achieve a power of 80% with a significance level of 0.05 for all the included appetite sensations and the ad libitum test meal, up to 108 participants per group would be required, depending on the specific outcome and effect size [30,49]. In addition to the low power of the study, the palatability differences observed between the test drinks present challenges when interpreting the results. Lastly, to determine whether the reduced desire to eat something sweet observed in the S&SEs group translates into decreased consumption of sweet foods, future studies could incorporate an ad libitum test meal with a buffet-style approach. This would enable assessment of the effect of the test drink on sweet food choices, for example [50]. Finally, our results cannot be directly extrapolated to comparisons with caloric sweeteners. Thus, the effects observed are specific to comparisons with a non-sweet, non-caloric control (water).

## 5. Conclusions

Ace-K/Cyc compared to water reduced the desire to eat something sweet acutely and after weight loss/maintenance periods, an effect that persisted after adjusting for taste differences. Effects on prospective consumption were also observed but were partially explained by palatability differences. Importantly, Ace-K/Cyc had similar effects on ad libitum energy intake to those of water, suggesting no adverse impact on energy balance. Due to the low sample size and power, larger studies are warranted to confirm these results.

## Figures and Tables

**Table 1:** Nutritional composition of the standardized breakfast and test drinks.

	Standardized Breakfast	Test Drinks		Ad Libitum Meal	
Served to	All Participants	S&SEs Group	Sugar Group	All Participants	
				Males	Females
Product	Arla Cultura^®^ with Oat and Cranberries ^1^	Atwell^®^0-Calories^®^ with Ace-K/Cyc	-	Ristorante Pizza Mozzarella	Ristorante Pizza Mozzarella
Amount (g)	340	5		1110	740
Energy (kJ)	1476			10,665	7110
Fat (g) - Saturated fat (g)	8.8 3.7	00		117.0 54.0	78.0 36.0
Carbohydrates (g) - Sugar (g)	40.8 24.8	00		246.0 22.8	164.0 15.2
Fiber (g)	13.6	0		13.8	9.2
Protein (g)	18.0	0		117.0	78.0
Salt (g)	0.34	0		10.8	7.2
Water (g)	250	400	405	250	250

^1^ Nutritional content of products (Arla Cultura^®^ with oat and cranberries, Ristorante Pizza Mozzarella, and Atwell^®^) as written on the packaging.

**Table 2:** Baseline characteristics at month 0 (test day 1).

	S&SEs Group (n = 15)	Sugar Group (n = 11)	Total (n = 26)	*p*-Value
Sex				
Female (n (%))	11 (73)	8 (73)	19 (73)	0.97
Male (n (%))	4 (27)	3 (27)	7 (27)	
Age (y)	48 ± 11	52 ± 8	49 ± 10	0.27
Height (cm)	172.3 ± 7.3	167.4 ± 8.0	170.2 ± 7.9	0.12
Weight (kg)	97.6 ± 14.1	89.7 ± 11.4	94.2 ± 13.4	0.13
BMI (kg/m^2^)	33.0 ± 5.0	32.0 ± 3.4	32.6 ± 4.3	0.56
Smoking status				
Non-smoker (n (%))	12 (80)	9 (82)	21 (81)	
Occasional smoker (n (%))	1 (7)	1 (9)	2 (8)	
Daily smoker (n (%))	2 (13)	1 (9)	3 (11)	0.93

Age, height, and smoking status measured at screening in the main study. Fasting body weight measured on test day 1 in the sub-study. Numerical data shown as means ± SD. *T*-tests were used to test for differences between intervention groups for all numerical outcomes, while categorical data were tested via logistic regression. No differences were found (all *p* > 0.05). Abbreviations: BMI, body mass index; SD, standard deviation; S&SEs, sweeteners and sweetness enhancers; y, year.

## Data Availability

The original contributions presented in the study are included in the article, further inquiries can be directed to the corresponding author.
